# PEDV NSP8 inhibits IFN-III production induced by MAVS through downregulation of PEX13

**DOI:** 10.1128/mbio.02396-25

**Published:** 2025-11-04

**Authors:** Jinxiu Lou, Huixin Zhu, Zhen Yang, Ping Jiang, Gongguan Liu, Xianwei Wang

**Affiliations:** 1 Key Laboratory of Animal Disease Diagnostics and Immunology, Ministry of Agriculture, MOE International Joint Collaborative Research Laboratory for Animal Health & Food Safety, College of Veterinary Medicine, Nanjing Agricultural University 70578, Nanjing, China; 2 Jiangsu Co-Innovation Center for Prevention and Control of Important Animal Infectious Diseases and Zoonoses, Yangzhou, China; Huazhong Agricultural University, Wuhan, Hubei, China

**Keywords:** PEDV, NSP8, PEX13, pexophagy, MAVS, type Ⅲ interferon

## Abstract

Porcine epidemic diarrhea virus (PEDV), an enteropathogenic coronavirus that causes severe intestinal disease in piglets, employs sophisticated strategies to subvert host antiviral immunity. While type III interferons (IFN-III) play a pivotal role in mucosal defense at intestinal epithelial barriers, the mechanisms underlying PEDV evasion of IFN-III signaling remain poorly understood. Given that peroxisomes serve as critical platforms for IFN-III signaling and that their abundance influences immune activation, we investigated the role of pexophagy in PEDV-mediated immune evasion. We demonstrated that the PEDV nonstructural protein NSP8 functions as a potent inhibitor of mitochondrial antiviral-signaling protein (MAVS)-dependent IFN-III production. Functional analyses revealed that NSP8 significantly reduces peroxisomal protein levels and promotes pexophagy. Mechanistically, mass spectrometry identified a direct interaction between NSP8 and PEX13. NSP8 also induces dose-dependent degradation of PEX13 via the autophagy-lysosomal pathway. This downregulation of PEX13 triggers ubiquitination of the peroxisomal import receptor PEX5, facilitating its recognition by the autophagy receptor NBR1 and the ubiquitin ligase PEX2, thereby promoting autophagic clearance of peroxisomes. Collectively, our findings reveal a novel immune evasion strategy in which PEDV exploits NSP8 to disrupt peroxisome homeostasis by targeting PEX13, thereby dismantling MAVS-dependent IFN-III antiviral signaling through pexophagy.

**IMPORTANCE:**

Porcine epidemic diarrhea virus (PEDV) NSP8 is a highly conserved protein that plays a crucial role in viral replication. Investigating the functional mechanisms of NSP8 contributes to a deeper understanding of PEDV pathogenesis and supports the development of antiviral strategies against coronaviruses. In this study, we elucidate how NSP8 suppresses type Ⅲ interferon (IFN-Ⅲ) production by promoting pexophagy through the downregulation of PEX13. We demonstrate that NSP8 directly interacts with PEX13 and enhances the ubiquitination of PEX5, leading to reduced peroxisome abundance and impaired mitochondrial antiviral-signaling protein (MAVS)-mediated IFN-Ⅲ signaling. These findings suggest that NSP8 hijacks the PEX13-dependent pexophagy pathway as a means of evading host antiviral defenses. This work provides critical insights into the interplay between viral proteins and host cellular machinery and highlights the NSP8-PEX13 axis as a promising target for therapeutic interventions aimed at enhancing antiviral immunity against PEDV and related coronaviruses.

## INTRODUCTION

Porcine epidemic diarrhea virus (PEDV), a member of the genus *Alphacoronavirus* in the *Coronaviridae* family, is one of the primary pathogens causing severe diarrhea in piglets, characterized by high morbidity and mortality (1). The PEDV genome is approximately 28 kb in length and encodes two polyproteins (PP1a and PP1ab), one accessory protein (open reading frame 3, ORF3), and four structural proteins: spike (S), envelope (E), membrane (M), and nucleocapsid (N) (2, 3). The innate immune response serves as the host’s first line of defense against viral infection. Consequently, many viruses, including PEDV, have evolved sophisticated strategies to modulate or evade host innate immunity during infection (4, 5). Previous studies have reported that the NSP8 of severe acute respiratory syndrome coronavirus 2 and porcine deltacoronavirus mediate evasion of the innate immune response during infection (6–8). However, the role of PEDV NSP8 in regulating host immunity remains unexplored.

Innate immune recognition of pathogen-associated molecular patterns triggers the production of interferons (IFNs), key antiviral cytokines that mediate host defense against viral infections (9, 10). Among them, type III interferons (IFN-λ/IFN-III) play a critical role in mucosal immunity (11), particularly at epithelial barriers, which serve as primary replication sites of PEDV. The mitochondrial antiviral-signaling protein (MAVS) acts as a central adaptor in the RIG-I-like receptor (RLR) pathway, transmitting signals from viral RNA sensors to promote IFN-III production (9, 12). Notably, MAVS is localized not only to mitochondria but also to peroxisomes, membrane-bound organelles traditionally known for lipid metabolism and reactive oxygen species (ROS) detoxification (13, 14). Emerging evidence suggests that peroxisome-associated MAVS contributes to the rapid induction of IFN-III and enhances antiviral defenses (9, 12). However, viruses have evolved strategies to counteract these responses, and PEDV encodes multiple nonstructural proteins (NSPs) that antagonize IFN production (15, 16). While NSP8 is implicated in PEDV replication and immune evasion, its precise role and mechanism remain poorly defined. In particular, it is unclear how PEDV manipulates peroxisome-mediated innate immune signaling to facilitate immune escape.

Autophagy, a conserved cellular degradation pathway that plays dual roles during viral infection: it can eliminate viral components (virophagy) or be exploited by viruses to degrade host immune regulators (17, 18). The selective autophagic degradation of peroxisomes, termed pexophagy, is tightly regulated by proteins, such as PEX13, a peroxisomal membrane protein essential for peroxisome biogenesis and maintenance (19–21). Although autophagy is known to intersect with innate antiviral signaling, the specific interplay between viral proteins, pexophagy, and IFN-III suppression remains unexplored.

In this study, we uncover a novel immune evasion mechanism in which PEDV NSP8 disrupts MAVS-dependent IFN-III signaling pathway by promoting peroxisome degradation. We demonstrated that NSP8 directly interacts with PEX13, downregulates its expression, and induces pexophagy, thereby impairing peroxisomal MAVS-mediated IFN-III production. These findings not only identify a unique immune evasion strategy employed by PEDV but also underscore the critical role of peroxisome homeostasis in antiviral innate immunity. Here, we aimed to determine whether, and how, PEDV NSP8 modulates peroxisome dynamics to inhibit the production of IFN-III.

## RESULTS

### PEDV inhibits the antiviral innate immune response

**Fig 1:**
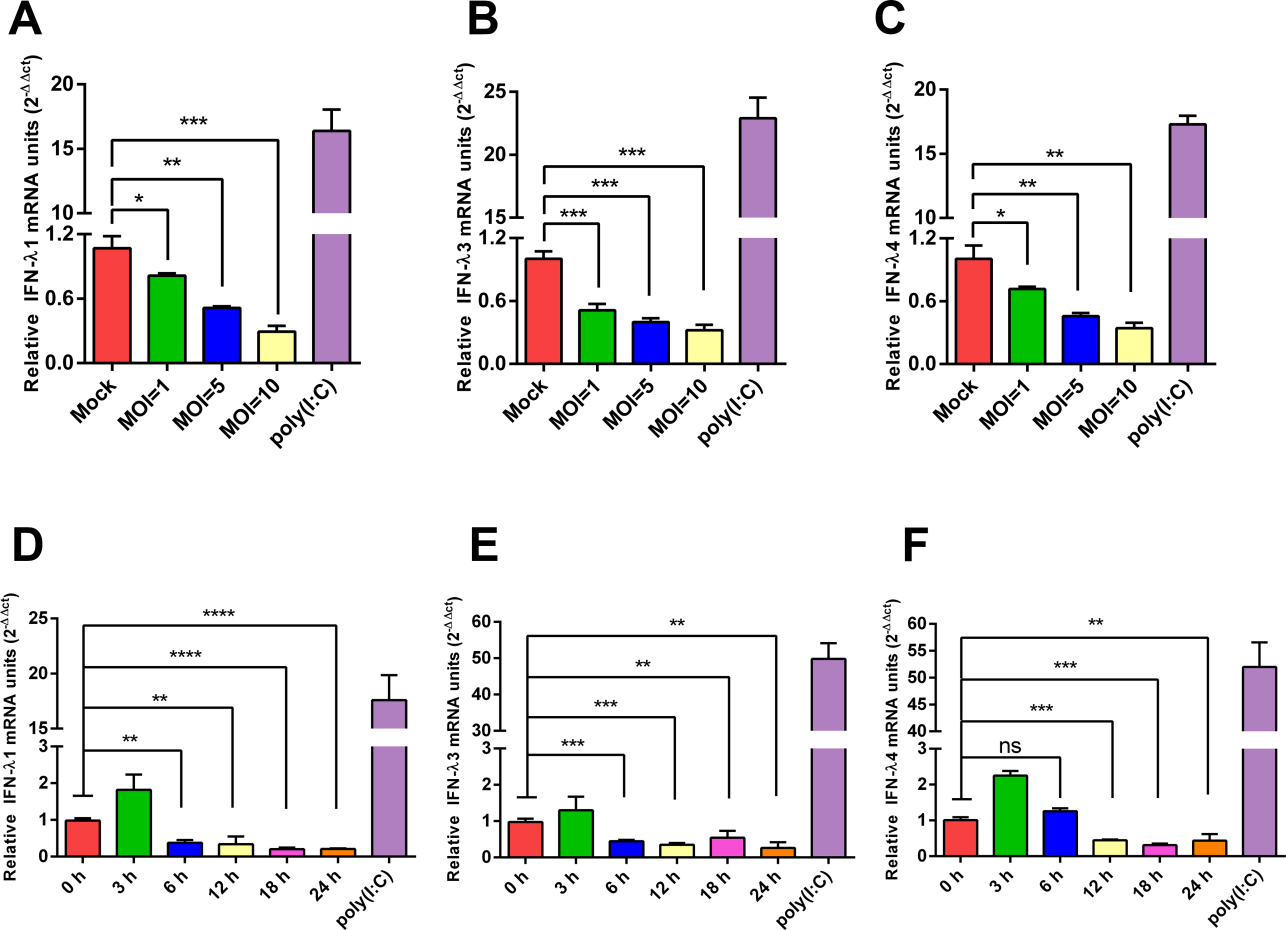
PEDV inhibits the antiviral innate immune response. (**A–C**) PEDV significantly suppressed the production of type III interferons (IFN-λ1, IFN-λ3, and IFN-λ4) in IPEC-J2 cells. Cells were infected with PEDV at different MOIs, and total RNA was extracted at 24 hpi. mRNA levels of IFN-λ subtypes were quantified by RT-qPCR. Cells treated with poly(I:C) (0.5 µg/mL) for 12 h served as a positive control. Date are means ± SDs from three independent experiments. *, *P* < 0.05; **, *P* < 0.01; ***, *P* < 0.001. (**D–F**) PEDV suppressed IFN-III production at the late stage of infection. IPEC-J2 cells were infected with PEDV at an MOI = 10, and total RNA was extracted at the indicated time points. IFN-λ1, IFN-λ3, and IFN-λ4 expression levels were determined by RT-qPCR. Poly(I:C)-treated cells served as the positive control. Date are means ± SDs from three independent experiments. *, *P* < 0.05; **, *P* < 0.01; ***, *P* < 0.001; ****, *P* < 0.0001; ns: not significant (*P* >0.05).

PEDV employs several strategies to inhibit the production of IFN to circumvent host antiviral defenses, facilitating rapid viral replication (5, 22, 23). The IFN-III family includes IFN-λ1, λ2, λ3, and λ4; however, Zhang et al. reported a potential absence of IFN-λ2 expression in swine. Therefore, this study specifically quantified the mRNA levels of IFN-λ1, λ3, and λ4. To investigate the impact of PEDV on the antiviral innate immune response, IPEC-J2 cells were infected with PEDV, and whole-cell lysates were collected at 24 hours post-infection (hpi) for analysis by quantitative real-time PCR (RT-qPCR). The results showed that infection with PEDV at different multiplicities of infection (MOI) significantly suppressed the production of IFN-λ1, IFN-λ3, and IFN-λ4 in IPEC-J2 cells (Fig. 1A through C). To further explore the temporal effects of PEDV infection on IFN-III expression, IPEC-J2 cells were infected with PEDV (MOI = 10) and harvested at designated time points for RT-qPCR analysis. The results demonstrated a transient upregulation of IFN-III mRNA expression at 3 hpi, followed by a time-dependent suppression as the infection progressed (Fig. 1D through F). These findings indicate that PEDV infection ultimately inhibits IFN-III expression in intestinal epithelial cells.

### PEDV NSP8 inhibits MAVS-mediated production of IFN-III

**Fig 2:**
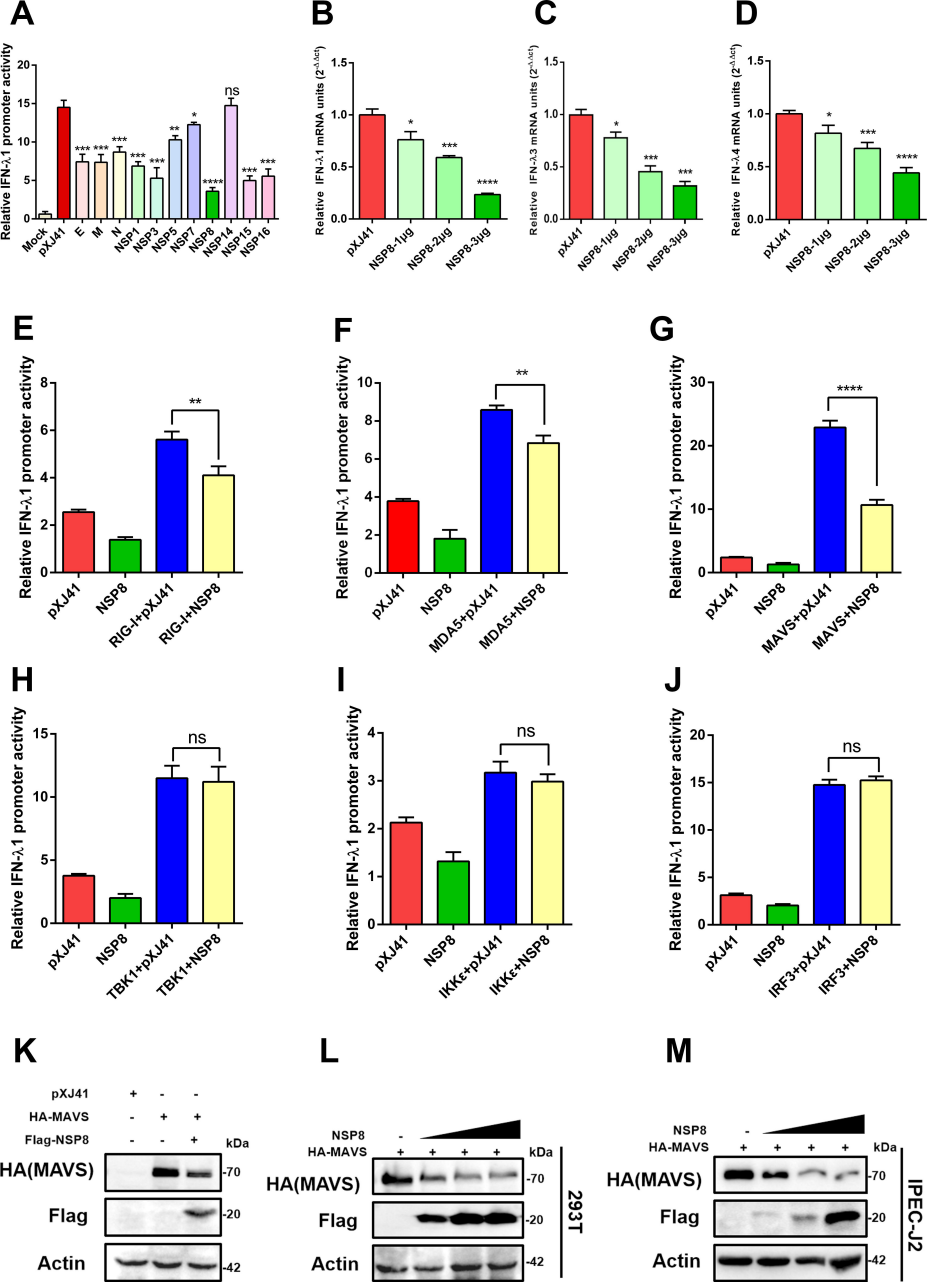
PEDV NSP8 inhibits MAVS-mediated the production of IFN-III. (**A**) Individual PEDV proteins were screened for suppression of the IFN-λ1 promoter using a luciferase assay. 293T cells were cotransfected with PEDV genes, IFN-λ1-Luc, and pRL-TK at a 2:1:0.2 ratio. At 24 hours post-transfection (hpt), cells except the mock group were stimulated with poly(I:C) (0.5 µg/mL) for 12 h. Date are means ± SDs from three independent experiments. *, *P* < 0.05; **, *P* < 0.01; ***, *P* < 0.001; ****, *P* < 0.0001; ns: not significant (P > 0.05). (**B–D**) NSP8 inhibited IFN-λ1, IFN-λ3, and IFN-λ4 mRNA expression in a dose-dependent manner. IPEC-J2 cells were transfected with increasing amounts of Flag-NSP8-expressing plasmids and stimulated with poly(I:C) prior to RT-qPCR. Date are means ± SDs from three independent experiments. *, *P* < 0.05; **, *P* < 0.01; ***, *P* < 0.001; ****, *P* < 0.0001. (**E–J**) NSP8 inhibited the activation of IFN-III mediated by MAVS. 293T cells were cotransfected with an active form of RIG-I (**E**), MDA5 (**F**), MAVS (**G**), TBK1 (**F**), IKKε (**I**), or IRF3 (**J**), along with the NSP8 gene and the IFN-λ1-Luc reporter at a ratio of 2:1:0.2 for 24 h. Cell lysates were prepared to measure luciferase activities. Date are means ± SDs from three independent experiments. *, *P* < 0.05; **, *P* < 0.01; ***, *P* < 0.001; ****, *P* < 0.0001; ns: not significant (*P* > 0.05). (**K**) NSP8 reduced MAVS protein expression. 293T cells were cotransfected with HA-MAVS and either an empty vector or Flag-NSP8. Western blotting was performed using anti-HA, anti-Flag, and anti-actin antibodies. (**L–M**) PEDV NSP8 degrades MAVS in a dose-dependent manner. 293T cells (**L**) or IPEC-J2 cells (**M**) seeded in 6-well plates were transfected with HA-MAVS (3 µg) together with empty vector or increasing doses of Flag-NSP8 (3, 4, or 5 µg), respectively. At 24 hpt, the cells were harvested for western blotting analysis with antibodies against HA-tag, Flag-tag, and actin.

To determine which PEDV proteins are involved in the regulation of IFN-III signaling, cells were co-transfected with an IFN-λ1 luciferase reporter construct and expression plasmids encoding individual viral proteins known to contribute to immunosuppression. A dual-luciferase reporter assay was then performed to evaluate changes in IFN-λ1 promoter activity. The results demonstrated that nearly all tested PEDV proteins significantly inhibited the production of IFN-III, with the exception of NSP14. Notably, NSP8 exhibited the most potent inhibitory effect (Fig. 2A). Therefore, the role of NSP8 was selected for further investigation in this study.

To further determine whether NSP8 is involved in regulating the antiviral innate immune response, varying amounts of Flag-NSP8 were transfected into IPEC-J2 cells for 24 h and then harvested for RT-qPCR analysis. The results showed that NSP8 suppressed the mRNA expression levels of IFN-λ1, IFN-λ3, and IFN-λ4 (Fig. 2B through D). To explore how NSP8 attenuates IFN-III-mediated antiviral responses, we examined its effect on key components of the RLRs, specifically RIG-I, MDA5, MAVS, TBK1, IRF3, and IKKε by evaluating their ability to activate IFN-λ1 promoter activity in the presence or absence of NSP8. While these signaling molecules individually activated the IFN-λ1 promoter, co-expression with NSP8 markedly inhibited MAVS-mediated activation, exhibited only weak suppression of RIG-I and MDA5-mediated activation, and had no inhibitory effect on TBK1-, IRF3-, or IKKε-induced IFN-λ1-luciferase activity (Fig. 2E through J). To further confirm the regulatory effect of NSP8 on MAVS expression, 293T cells were co-transfected with Flag-NSP8 and HA-MAVS for 24 h, followed by a western blotting assay. Immunoblot analysis demonstrated that NSP8 significantly reduced MAVS protein levels (Fig. 2K). Additionally, co-transfection of 293T and IPEC-J2 cells with a fixed amount of HA-MAVS and increasing doses of Flag-NSP8 plasmid revealed that NSP8 inhibited MAVS expression in a dose-dependent manner (Fig. 2L and M). Collectively, these findings suggest that PEDV NSP8 suppresses type III IFN production by downregulating MAVS-mediated signaling.

### NSP8 reduces peroxisome abundance

**Fig 3:**
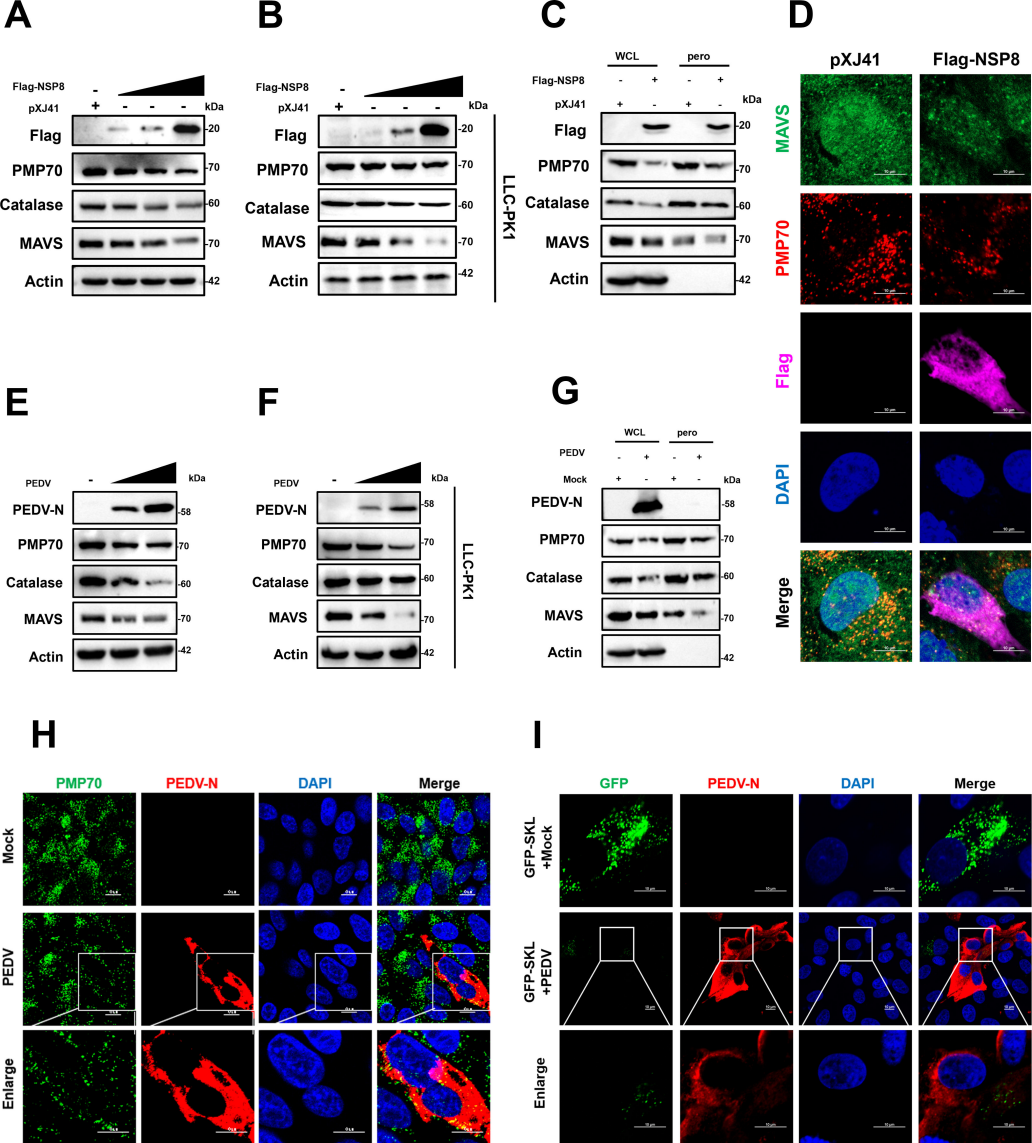
NSP8 reduces peroxisome abundance. (**A–B**) PEDV NSP8 degrades peroxisome-associated proteins PMP70, catalase, and MAVS in a dose-dependent manner. IPEC-J2 cells and LLC-PK1 cells seeded in 6-well plates were transfected with either the empty vector or increasing doses of Flag-NSP8 (4, 5, or 6 µg). At 24 hpt, the cells were harvested for western blotting analysis with antibodies against Flag-tag, PMP70, catalase, MAVS, and actin. (**C**) Whole cell lysate or peroxisomes were isolated from pxj41-transfected or Flag-NSP8-transfected IPEC-J2 cells and analyzed by western blotting using the indicated antibodies. (**D**) Reduction in the abundance of peroxisomes in NSP8-transfected cells. IPEC-J2 cells grown on coverslips were transfected with Flag-NSP8 or the pXJ41 empty vector for 24 h and then incubated with anti-PMP70 (peroxisome marker) antibody, anti-MAVS antibody, and anti-Flag antibody for immunostaining. (**E–F**) PEDV degrades peroxisome-associated proteins PMP70 and catalase in a dose-dependent manner. IPEC-J2 cells and LLC-PK1 cells seeded in 6-well plates were infected with PEDV. At 24 hpi, the cells were harvested for western blotting analysis with antibodies against PEDV-N, PMP70, catalase, MAVS, and actin. (**G**) Whole cell lysate or peroxisomes were isolated from mock-infected or PEDV-infected IPEC-J2 cells and analyzed by western blotting using the indicated antibodies. (**H**) Reduction in the abundance of peroxisomes in PEDV-infected cells. IPEC-J2 cells grown on coverslips were infected with PEDV for 24 h and then incubated with anti-PMP70 (peroxisome marker) antibody and anti-PEDV N antibody for immunostaining. (**I**) IPEC-J2 cells were transfected with GFP-SKL for 24 h, and then samples were collected at 24 hpi with PEDV, followed by incubation with anti-PEDV N antibody for immunostaining.

A previous study reported that IFN-III-mediated antiviral signaling in intestinal epithelial cells primarily depends on peroxisomes (12). To assess the influence of NSP8 on the expression of peroxisome-related proteins, peroxisomal membrane protein 70 (PMP70) and catalase were used as peroxisomal markers (24). IPEC-J2 cells and LLC-PK1 cells were transfected with varying amounts of Flag-NSP8. The results showed that NSP8 decreased the expression of endogenous PMP70, catalase, and MAVS in a dose-dependent manner in IPEC-J2 cells and LLC-PK1 (Fig. 3A and B). Therefore, we speculated that NSP8 induces the degradation of peroxisomes, leading to a decrease in MAVS localized in peroxisomes. To test this hypothesis, the peroxisomes were extracted from IPEC-J2 cells transfected with pXJ41 or Flag-NSP8. The results confirmed that NSP8 reduces the concentration of MAVS and related proteins on peroxisomes (Fig. 3C). To further investigate the effect of NSP8 on peroxisome abundance, IPEC-J2 cells were transfected with Flag-NSP8, and the numbers and morphology of peroxisomes were examined. In cells transfected with the empty vector pXJ41, peroxisomes were abundantly distributed throughout the cytoplasm. In contrast, the number of peroxisomes and MAVS located on peroxisomes was significantly reduced in NSP8-transfected cells (Fig. 3D). These results indicate that NSP8 significantly reduces peroxisome abundance in cells.

To further explore whether PEDV similarly modulates peroxisome abundance, IPEC-J2 cells and LLC-PK1 cells were infected with PEDV at different MOIs, and cell lysates were harvested at 24 hpi. The results demonstrated that the expression levels of endogenous PMP70, catalase, and MAVS were significantly decreased in PEDV-infected cells in a dose-dependent manner (Fig. 3E and F). To further investigate whether PEDV-mediated suppression of peroxisome-associated proteins and MAVS occurs specifically on peroxisomes, the peroxisomes were isolated from PEDV-infected IPEC-J2 cells. The results demonstrated that PEDV reduces the protein levels of MAVS and related proteins on peroxisomes (Fig. 3G). Finally, the abundance of peroxisomes in PEDV-infected IPEC-J2 cells was assessed. Confocal microscopy revealed a significant reduction in PMP70 expression in PEDV-infected cells compared with uninfected cells (Fig. 3H). Moreover, another peroxisome marker, GFP-Ser-Lys-Leu (SKL), was used to evaluate peroxisome number. The SKL motif, a peroxisomal matrix-targeting signal peptide, was fused to the C-terminus of GFP to direct it to peroxisomes. Consistent with PMP70 staining, GFP fluorescence was markedly reduced in PEDV-infected cells compared with uninfected controls (Fig. 3I). These results demonstrate that NSP8 suppresses MAVS by reducing the abundance of peroxisomes.

### PEDV NSP8 downregulates the production of IFN-III via the pexophagy pathway

**Fig 4:**
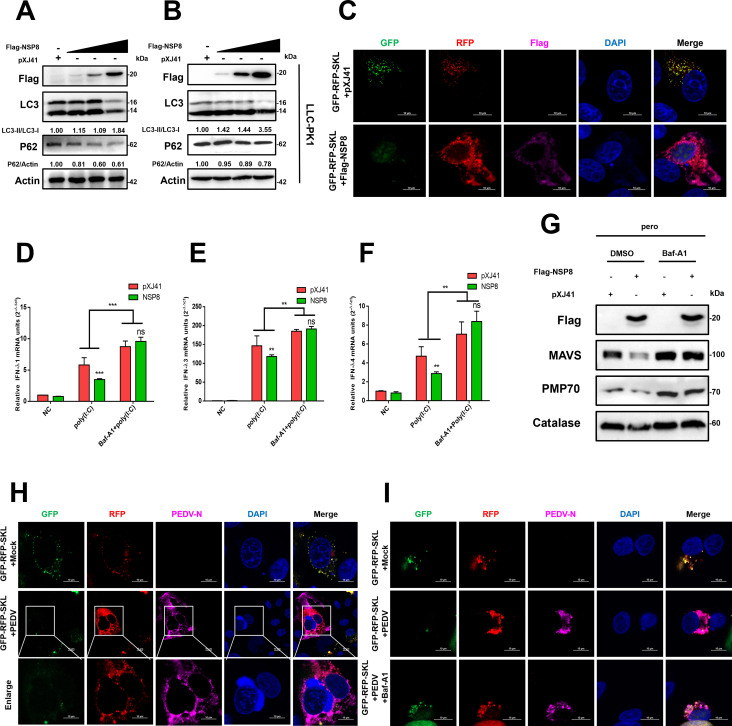
PEDV NSP8 downregulates the production of IFN-III via the autophagy pathway. (**A and B**) Ectopic expression of PEDV NSP8 induces autophagy. IPEC-J2 cells and LLC-PK1 cells seeded in 12-well plates were transfected with Flag-NSP8 (2, 2.5, 3 µg). At 24 hpt, the cells were harvested for western blotting analysis with antibodies against LC3, P62, Flag-tag, and actin. (**C**) IPEC-J2 cells were co-transfected with GFP-RFP-SKL and Flag-NSP8 or pXJ41 for 24 h, followed by incubation with anti-Flag antibody for immunostaining. (**D–F**) Baf-A1 can reverse the suppression of IFN-III production by NSP8. IPEC-J2 cells were transfected with pXJ41 (2 µg) or Flag-NSP8 (2 µg). After 8 h, cells were DMSO-treated or treated with Baf-A1 (25 nM). At 24 hpt, the cells were subsequently stimulated with 0.5 µg/mL poly(I:C) for 12 h before harvesting for RT-qPCR analysis. Date are means ± SDs from three independent experiments. *, *P* < 0.05; **, *P* < 0.01; ***, P < 0.001; ns: not significant (*P* > 0.05).(**G**) Baf-A1 reverses NSP8-mediated suppression of MAVS on peroxisomes in IPEC-J2 cells. IPEC-J2 cells were transfected with pXJ41 or Flag-NSP8. After 8 h, cells were DMSO-treated or treated with Baf-A1. After 24 h, the cells were harvested, and peroxisomes were extracted using a kit. Subsequently, western blotting was performed with the corresponding antibodies for analysis. (**H**) IPEC-J2 cells were transfected with GFP-RFP-SKL for 24 h, and then samples were collected at 24 hpi with PEDV, followed by incubation with anti-PEDV N antibody for immunostaining. (**I**) IPEC-J2 cells were transfected with GFP-RFP-SKL for 24 h, then mock-infected or infected with PEDV. Cells were treated with DMSO or Baf-A1 (25 nM) at 2 hpi with PEDV. After another 22 hpi, cells were incubated with anti-PEDV N antibody for immunostaining.

A previous study reported that the reduction in peroxisome abundance is primarily regulated through selective autophagy, specifically pexophagy (25). To investigate whether NSP8-induced peroxisome reduction is associated with pexophagy, IPEC-J2 cells and LLC-PK1 cells were transfected with increasing amounts of Flag-NSP8 and analyzed by western blotting to assess the expression of autophagy markers LC3 and p62. The results demonstrated that a dose-dependent increase in the LC3-II/LC3-I ratio and a concurrent decrease in p62 levels in both cell types (Fig. 4A and B) suggested that NSP8 activates the autophagy pathway. To further determine whether NSP8 specifically induces pexophagy, cells were co-transfected with either the empty vector (pXJ41) or Flag-NSP8 and the RFP-GFP-SKL reporter construct, a dual-fluorescence probe targeting peroxisomes via the PTS1 signal. Confocal microscopy was employed to monitor changes in GFP fluorescence, which is quenched in acidic lysosomes, while RFP remains stable. In cells transfected with Flag-NSP8, the GFP signal was significantly reduced, resulting in fewer overlapping GFP/RFP signals (Fig. 4C). This result indicated that NSP8 promotes peroxisome degradation via pexophagy. To determine whether NSP8-induced suppression of IFN-III is mediated via pexophagy, the autophagy inhibitor Bafilomycin A1 (Baf-A1) was applied to pXJ41 or Flag-NSP8-transfected cells. RT-qPCR results demonstrated that Baf-A1 reversed the NSP8-induced suppression of IFN-III (IFN-λ1, IFN-λ3, and IFN-λ4) expression (Fig. 4D through F). Finally, to determine whether NSP8-mediated inhibition of MAVS occurs through pexophagy, IPEC-J2 cells transfected with either pXJ41 or Flag-NSP8 were treated with Baf-A1, and then the peroxisomes were isolated. The results demonstrated that Baf-A1 alleviated NSP8-induced suppression of MAVS in peroxisomes (Fig. 4G).

Autophagy and the ubiquitin-proteasome system are the two major intracellular degradation pathways. While individual proteins are typically degraded via the proteasome, organelles, such as peroxisomes, are degraded via the lysosomal pathway. To assess which pathway mediates peroxisome degradation during PEDV infection, IPEC-J2 cells were treated with the proteasome inhibitor MG132 or the autophagy inhibitors Baf-A1 and 3-MA. In both mock- and PEDV-infected cells, degradation of PMP70 and catalase was unaffected by MG132 but was blocked by Baf-A1 and 3-MA (Fig. S1), confirming that PEDV-induced peroxisome degradation is mediated by autophagy. To further validate whether PEDV infection induces pexophagy, the RFP-GFP-SKL reporter was transfected into PEDV-infected cells. Confocal microscopy revealed that the GFP fluorescence was quenched in acidic lysosomes, resulting in fewer overlapping GFP/RFP signals. Many PEDV-infected cells displayed RFP signals that did not co-localize with GFP, indicative of active pexophagy (Fig. 4H). Importantly, treatment with Baf-A1 rescued the suppression of the RFP/GFP ratio in PEDV-infected IPEC-J2 cells (Fig. 4I). Taken together, these findings suggest that PEDV NSP8 suppresses the expression of MAVS on peroxisomes and the production of IFN-III by promoting peroxisome degradation via pexophagy.

### PEDV NSP8 interacts with PEX13

**Fig 5:**
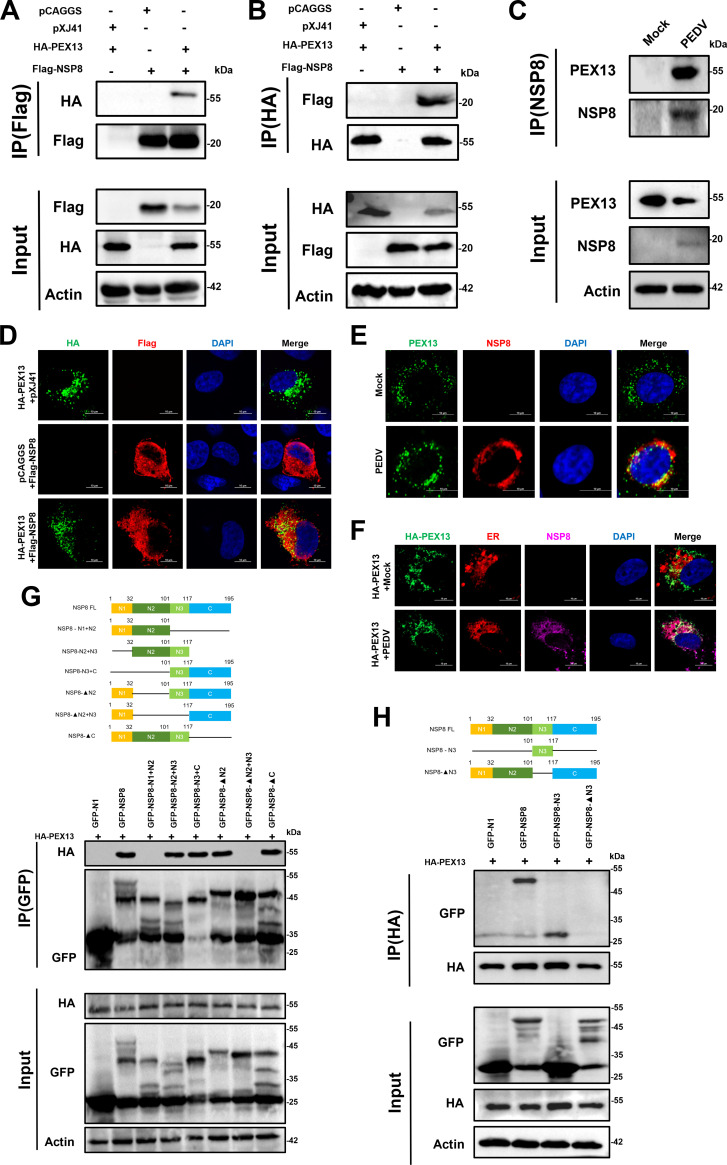
PEDV NSP8 interacts with PEX13. (**A and B**) PEDV NSP8 interacts with PEX13. PEDV NSP8 interacts with PEX13. IPEC-J2 cells seeded in 60 mm dishes were transfected with Flag-NSP8 (8 µg) or an empty vector (8 µg) for 6 h, followed by medium replacement. After an additional 6 h, the cells underwent secondary transfection with HA-PEX13 (4 µg) or an empty vector (4 µg) for 6 h, after which the medium was refreshed again. At 36 hpt, cells were processed for IP using either anti-Flag magnetic beads (**A**) or anti-HA magnetic beads (**B**). Both whole-cell lysates (input) and immunoprecipitated proteins were immunoblotted with antibodies targeting Flag-tag, HA-tag, and actin. (**C**) IPEC-J2 cells seeded in 60 mm dishes were infected with PEDV or mock. At 24 hpi, cells were processed for IP using anti-NSP8. Both input and immunoprecipitated proteins were immunoblotted with antibodies targeting NSP8, PEX13, and actin. (**D**) PEDV NSP8 colocalizes with PEX13 in the cytoplasm. IPEC-J2 cells were sequentially transfected with Flag-NSP8 (4 µg) along with either the empty vector or HA-PEX13 (2 µg). At 36 hpt, cells were stained with anti-HA (green) and anti-Flag (red) antibodies and subjected to confocal microscopy. Nuclei were stained with DAPI (blue) (scale bar: 10 µm). (**E**) IPEC-J2 cells were sequentially infected with PEDV or mock. At 24 hpi, cells were stained with anti-PEX13 (green) and anti-NSP8 (red) antibodies and subjected to confocal microscopy. Nuclei were stained with DAPI (blue) (scale bar: 10 µm). (**F**) Cells transfected with HA-PEX13 and infected with PEDV (MOI = 10) for 12 h were fixed and probed with mouse anti-NSP8 antibody (purple), ER-Tracker Red (red), and rabbit anti-HA antibody (green), then observed by confocal microscopy. Bars = 10 µm. (**G**) PEX13 interacts with NSP8-N3 (102-117 aa). 293T cells seeded in 60 mm dishes were sequentially transfected with the indicated plasmids (GFP-N1 empty vector [8 µg], GFP-NSP8-FL [8 µg], GFP-NSP8-N1+N2 [8 µg], GFP-NSP8-N2+N3 [8 µg], GFP-NSP8-N3+C [8 µg], GFP-NSP8-▲N2 [8 µg], GFP-NSP8-▲N2+N3 [8 µg], or GFP-NSP8-▲C [8 µg]), and HA-PEX13 (4 µg). At 36 hpt, the cells were processed for IP with anti-GFP magnetic beads. Whole-cell lysates (WCLs) and precipitated proteins were probed with antibodies against GFP-tag, HA-tag, and actin. (**H**) 293T cells were subjected to staggered transfection with specified plasmids (GFP-N1 empty vector [8 µg], GFP-NSP8-FL [8 µg], GFP-NSP8-N3 [8 µg], or GFP-NSP8-▲N3 [8 µg]) in combination with HA-PEX13 (4 µg). At 36 hpt, cells were processed for IP with anti-HA magnetic beads. Input and precipitated proteins were probed with antibodies against Flag-tag, HA-tag, and actin.

To identify host proteins involved in NSP8-triggered pexophagy, a mass spectrometry (MS)-based proteomic screening was conducted. Notably, PEX13, a key component of the peroxisomal matrix protein import machinery (26, 27), was the only peroxisome-associated protein significantly enriched in NSP8 immunoprecipitates. To validate the results of MS, a co-immunoprecipitation (Co-IP) assay was performed. 293T cells were co-transfected with Flag-NSP8 and pCAGGS-HA-PEX13 plasmids, followed by immunoprecipitation using anti-Flag antibodies. The Co-IP results confirmed that NSP8 interacts with PEX13 (Fig. 5A). A reverse Co-IP experiment was subsequently performed using anti-HA antibodies, which further demonstrated that NSP8 could be efficiently co-immunoprecipitated with PEX13 (Fig. 5B). Together, the forward and reverse Co-IP results support a specific interaction between PEDV NSP8 and PEX13 under overexpression conditions. To assess whether this interaction occurs endogenously during PEDV infection, IPEC-J2 cells infected with PEDV (MOI = 10) for 12 hpi were subjected to Co-IP using anti-NSP8 antibodies. As shown in Fig. 5C, endogenous PEX13 was successfully co-immunoprecipitated with NSP8 in PEDV-infected cells, confirming that the interaction occurs in a physiological context. Confocal microscopy further supported this finding by demonstrating strong colocalization between NSP8 and PEX13 (Fig. 5D and E). As a coronavirus, PEDV utilizes NSP8 as a key component of its viral replication complex (NSP7/NSP8/NSP12/dsRNA), and NSP8 can bind RNA templates to initiate the synthesis of complementary oligonucleotides and exhibits secondary RNA-dependent RNA polymerase activity (28, 29). Therefore, the interaction between NSP8 and PEX13 may occur either within the context of the replication complex on endoplasmic reticulum (ER). To confirm this, the confocal microscopy was used to show whether HA-PEX13 colocalized with NSP8 in the ER. As shown in Fig. 5F, the PEX13 protein with NSP8 was located in the ER. As a negative control, in the mock-infected group, there was no co-localization between PEX13 and the ER. Therefore, these results hypothesize that NSP8 in the PEDV replication complex recruits PEX13 to form the ER-peroxisome membrane contact sites.

To map the domain of NSP8 responsible for the interaction, a series of NSP8 truncation mutants wase generated based on structural predictions using SWISS-MODEL. The tertiary structure of NSP8 was predicted to contain a long α-helix (N2), a random coil region (N3), and a short α-helix (C), with the remaining region designated as N1. Co-IP analysis showed that the N3 domain of NSP8 was responsible for the interaction with PEX13. Deletion of the N3 domain (GFP-NSP8-ΔN3) abolished this interaction (Fig. 5G and H), confirming the importance of this region in mediating binding.

### PEDV NSP8 induces pexophagy and inhibits IFN-III production by degrading PEX13

**Fig 6:**
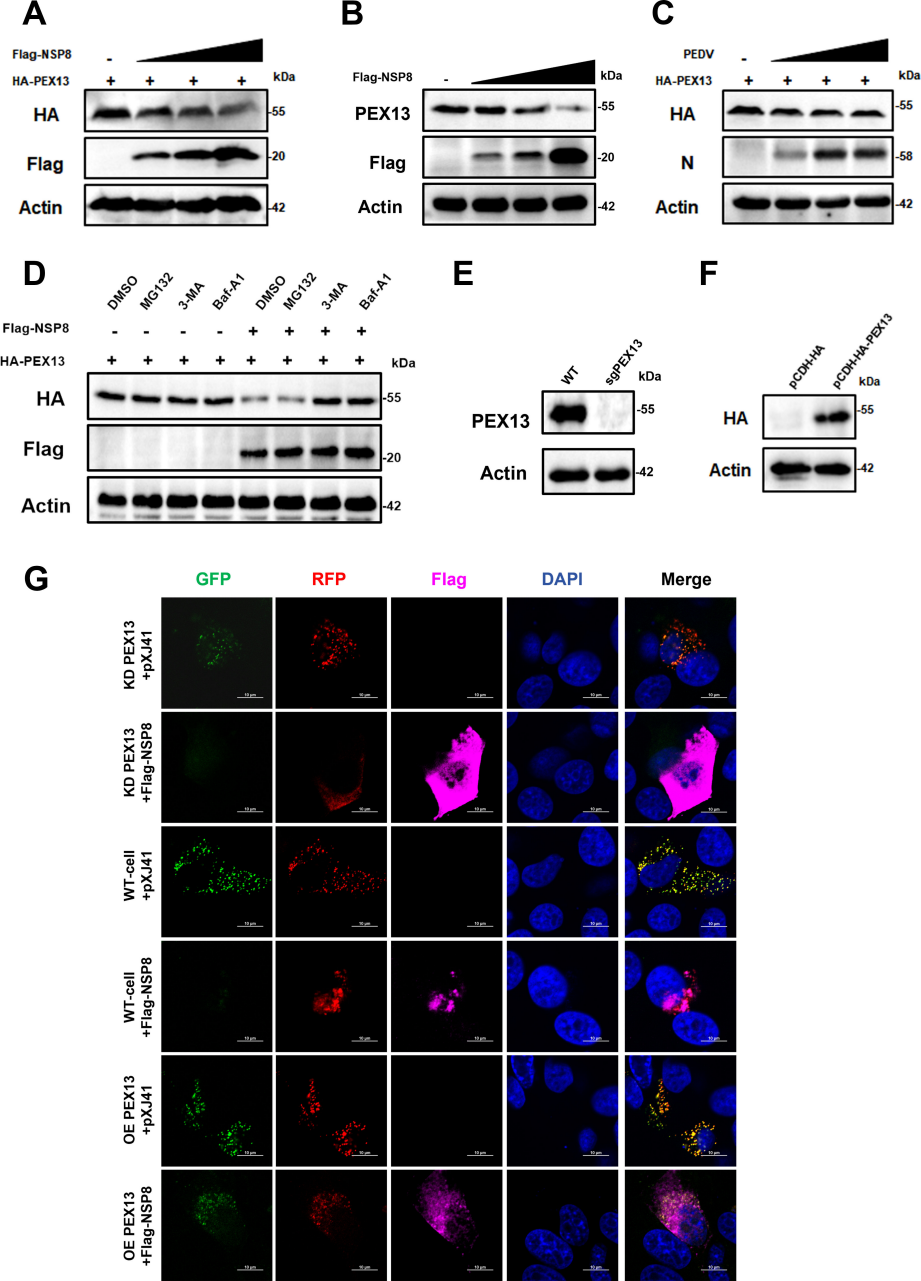
PEDV NSP8 induces pexophagy via PEX13. (**A**) PEDV NSP8 degrades PEX13 in a dose-dependent manner. IPEC-J2 cells seeded in 12-well plates were staggered-transfected with HA-PEX13 (1 µg) together with the empty vector pXJ41 or increasing doses of Flag-NSP8 (2, 2.5, or 3 µg). At 36 hpt, the cells were harvested for western blotting analysis using antibodies against HA-tag, Flag-tag, and actin. (**B**) IPEC-J2 cells were transfected with increasing doses of Flag-NSP8 (2, 2.5, or 3 µg). At 24 hpt, cells were collected for western blotting using antibodies against Flag-tag, PEX13, and actin. (**C**) PEDV degrades PEX13 in a dose-dependent manner. IPEC-J2 cells seeded in 12-well plates were transfected with HA-PEX13 (1 µg). At 24 hpt, the cells were infected with mock or increasing MOIs of PEDV (MOI = 1, 5, 10). At 24 hpi, the cells were harvested for western blotting analysis using antibodies against HA-tag, PEDV-N, and actin. (**D**) Effects of MG132, Baf-A1, and 3-MA on PEX13 degradation induced by NSP8. IPEC-J2 cells seeded in 12-well plates were staggered-transfected with HA-PEX13 (1 µg) and Flag-NSP8 (3 µg). At 12 hpt, cells were treated with DMSO, MG132 (10 nM), 3-MA (2 mM), or Baf-A1 (25 nM). After another 24 h, cells were harvested for western blotting analysis using antibodies against HA-tag, Flag-tag, and actin. (**E**) Western blotting analysis of lysates from IPEC-J2 cells infected with sgPEX13 or pLentiCRISPR-V2. β-actin was used as the loading control. (**F**) Western blotting analysis of lysates from IPEC-J2 cells infected with pCDH-HA-PEX13 or pCDH-HA. β-actin was used as the loading control. (**G**) NSP8-induced pexophagy is associated with the reduction of PEX13. IPEC-J2 WT, KD PEX13, or OE PEX13 cells were seeded in dishes and transfected with GFP-RFP-SKL along with either pXJ41 or Flag-NSP8. At 24 hpt, the cells were fixed and incubated with anti-Flag antibody for immunostaining.

To further investigate the relationship between NSP8 and PEX13, IPEC-J2 cells were transfected with a fixed amount of pCAGGS-HA-PEX13 and increasing amounts of Flag-NSP8. Western blotting revealed that NSP8 reduced the expression of overexpressed PEX13 in a dose-dependent manner (Fig. 6A). In a separate experiment, IPEC-J2 cells were transfected with increasing doses of Flag-NSP8, and endogenous PEX13 levels were analyzed by western blotting. As shown in Fig. 6B, NSP8 suppressed endogenous PEX13 expression in a dose-dependent manner. Similarly, PEDV infection also significantly decreased PEX13 expression in a dose-dependent manner (Fig. 6C). These results collectively demonstrated that NSP8 mediates the degradation of PEX13. Next, we explored the pathway responsible for NSP8-induced PEX13 degradation. As shown in Fig. 6D, treatment with the autophagy inhibitors 3-MA and Baf-A1 reversed the degradation of PEX13, whereas the proteasome inhibitor MG132 had no effect. This indicates that NSP8 promotes the degradation of PEX13 via the autophagy pathway.

Previous studies have demonstrated that depletion of PEX13 can trigger pexophagy (19, 20). To further investigate whether NSP8-induced pexophagy is dependent on the degradation of PEX13, we generated IPEC-J2 wild type (WT), PEX13 knockdown (KD), and PEX13 overexpression (OE) cell lines (Fig. 6E and F). These cells were transfected with either the empty vector pXJ41 or Flag-NSP8, along with the RFP-GFP-SKL reporter, and fixed at 24 h post-transfection. Confocal microscopy revealed that in WT cells, NSP8 overexpression significantly reduced the RFP/GFP signal ratio compared with vector-transfected controls. In PEX13-KD cells, the RFP/GFP ratio was further decreased, while in PEX13-OE cells, GFP signal quenching was absent (Fig. 6G). These results demonstrate that NSP8 induces pexophagy by downregulating PEX13 expression.

To evaluate the effect of the NSP8-PEX13 interaction on IFN-III signaling, increasing amounts of NSP8 were transfected into IPEC-J2-WT, IPEC J2-PEX13 KD, and IPEC-J2-PEX13 OE cells, and MAVS expression was assessed. NSP8 significantly suppressed MAVS expression in both WT and KD cells in a dose-dependent manner. However, this inhibitory effect was abolished in PEX13-OE cells (Fig. S2A). In addition, poly(I:C)-induced expression of IFN-λ1 was significantly higher in PEX13-OE cells compared to WT cells, while it was markedly reduced in PEX13-KD cells (Fig. S2B). Overall, these findings indicate that PEDV NSP8 suppresses MAVS expression and inhibits IFN-III production by promoting pexophagy through PEX13 degradation.

### NSP8 triggers pexophagy by downregulating PEX13 and inducing PEX5 ubiquitination

**Fig 7:**
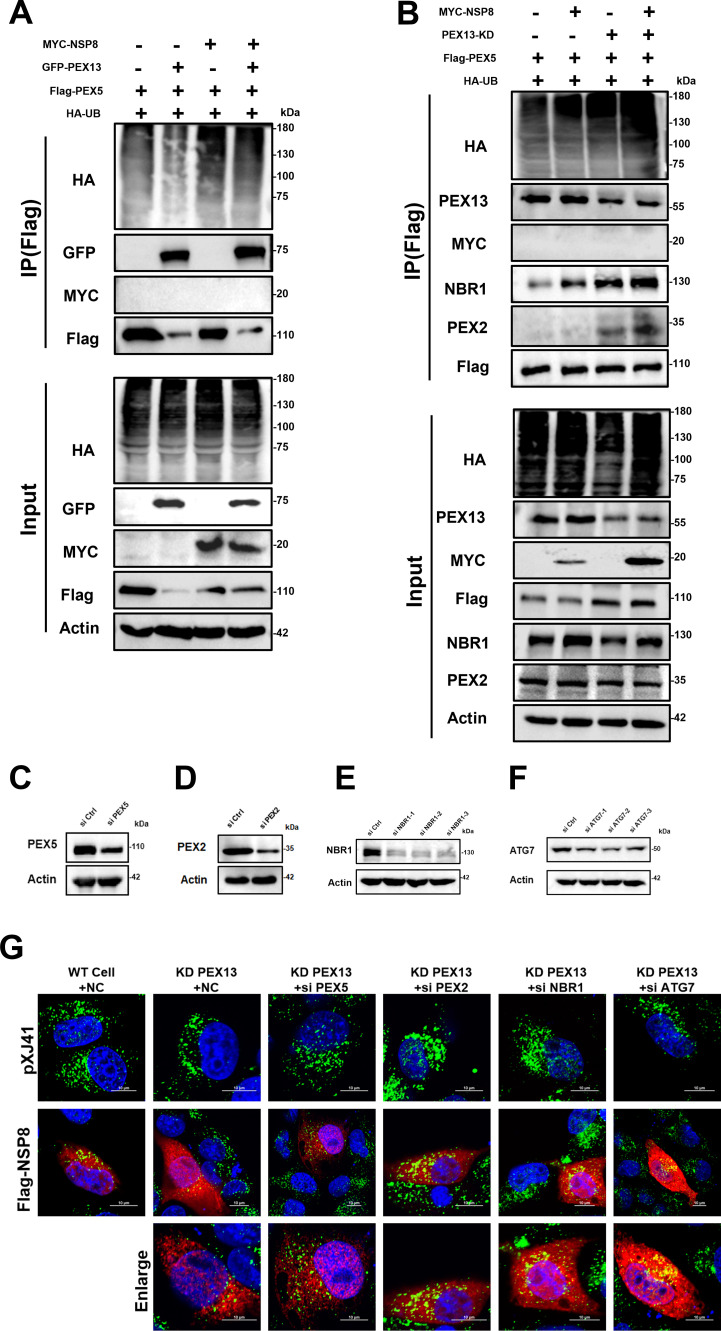
NSP8 triggers pexophagy through the downregulation of PEX13. (**A**) NSP8 partially alleviates the inhibitory effect of PEX13 on PEX5 ubiquitination. 293T cells were transfected with MYC-NSP8 (12 µg), GFP-PEX13 (4 µg), HA-Ub (6 µg), and Flag-PEX5 (6 µg). At 36 hpt, cells were processed for IP using anti-Flag magnetic beads. Input and precipitated proteins were probed using antibodies against Flag-tag, MYC-tag, HA-tag, GFP-tag, and actin. (**B**) NSP8 promotes the ubiquitination of PEX5 and enhances the interaction of PEX5 with NBR1 and PEX2 in PEX13 knockdown cells. WT or KD PEX13 cells were transfected with MYC-NSP8 (12 µg), Flag-PEX5 (6 µg), and HA-Ub (6 µg). At 36 hpt, cells were processed for IP with anti-Flag magnetic beads. Input and precipitated proteins were probed using antibodies against Flag-tag, MYC-tag, HA-tag, PEX13, NBR1, PEX2, and actin. (**C–F**) IPEC-J2 cells were transfected with siCtrl, siPEX5, siPEX2, siNBR1, or siATG7. At 36 hpt, cells were harvested for western blotting analysis using antibodies against actin, PEX5, PEX2, NBR1, or ATG7. (**G**) The NSP8-mediated reduction in peroxisome abundance was completely abolished in cells with knockdown (KD) of PEX5, PEX2, NBR1, or ATG7. Representative images of KD PEX13 or WT cells treated with the indicated siRNA and transfected with pXJ41 or Flag-NSP8 were immunostained for the peroxisomal marker PMP70 at 36 hpt.

Recent studies have demonstrated that depletion of PEX13 leads to the accumulation of ubiquitinated PEX5 on peroxisomal membranes, which in turn triggers ubiquitin-dependent pexophagy (30, 31). To explore whether NSP8 regulates PEX5 ubiquitination through its effects on PEX13, 293T cells were co-transfected with GFP-PEX13 or the empty vector, along with pXJ41-MYC-NSP8 or its vector control, HA-tagged ubiquitin (HA-UB), and Flag-tagged PEX5. As shown in Fig. 7A, overexpression of PEX13 significantly reduced the polyubiquitination of PEX5 protein. However, co-expression of NSP8 and PEX13 reversed this effect, enhancing PEX5 ubiquitination compared to PEX13 overexpression alone. Interestingly, while PEX5 was found to interact with PEX13, no direct interaction was observed between PEX5 and NSP8. These findings suggest that NSP8 relieves PEX13-mediated suppression of PEX5 ubiquitination. To further elucidate the mechanism underlying NSP8-induced pexophagy via ubiquitinated PEX5 accumulation, we performed a measure of three critical pexophagy-related proteins in PEX13-KD cells: NBR1, an autophagy receptor responsible for recognizing ubiquitinated peroxisomes and recruiting them to nascent autophagosomes; PEX2, a peroxisomal E3 ubiquitin ligase mediating peroxisomal protein ubiquitination during pexophagy induction; and PEX5, a well-characterized ubiquitination target in pexophagy. As demonstrated in Fig. 7B, knockdown of PEX13 significantly increased PEX5 ubiquitination, and this effect was further enhanced by NSP8 overexpression. Co-immunoprecipitation assays revealed that PEX5 interacted with both NBR1 and PEX2, and these interactions were strengthened in PEX13-KD cells compared with WT cells (Fig. 7B). Interference efficiency of siRNAs targeting PEX5, PEX2, NBR1 (siNBR1-2), and ATG7 (siATG7-2) was validated, and the most efficient siRNAs were selected for further experiments (Fig. 7C through F). ATG7, essential for autophagosome formation, served as a positive control for autophagy inhibition. To investigate whether these pexophagy-related proteins are involved in NSP8-mediated peroxisome loss due to PEX13 degradation, both WT and PEX13-KD IPEC-J2 cells were transfected with siRNAs targeting PEX2, PEX5, NBR1, and ATG7, followed by transfection with either Flag-NSP8 or the empty vector. Cells were fixed and analyzed by confocal microscopy to assess peroxisome abundance via PMP70 immunostaining. As expected, NSP8 reduced peroxisome abundance in both WT and PEX13-KD cells. However, silencing PEX2, PEX5, NBR1, or ATG7 significantly reversed the NSP8-induced loss of peroxisomes in PEX13-KD cells (Fig. 7G). Taken together, these data suggest that NSP8 triggers pexophagy by downregulating PEX13 expression, which in turn induces PEX5 ubiquitination and leads to the recruitment of pexophagy machinery, culminating in peroxisome degradation.

## DISCUSSION

PEDV is one of the primary pathogens responsible for diarrhea in piglets, characterized by high morbidity and mortality. The virus primarily invades pigs through the digestive tract, targeting small intestinal epithelial cells for replication. The intestinal mucosa mounts a type III interferon response as the first line of antiviral defense at mucosal surfaces (32, 33). However, PEDV evades host immunity, notably by suppressing IFN-III production. This study aimed to elucidate the molecular mechanisms underlying PEDV-mediated evasion of IFN-III responses, thereby identifying potential therapeutic targets for combating PEDV infection.

Given that PEDV causes acute intestinal disease, we hypothesized that it disrupts the innate immune defenses of intestinal epithelial cells, particularly IFN-III signaling. Consistent with this hypothesis, our data revealed that PEDV infection inhibits the production of IFN-III in IPEC-J2 cells. Mechanistically, PEDV NSP8 acts as a potent immune suppressor, inhibiting the promoter activity of IFN-III by downregulating the expression of MAVS. Both type I and type III interferons are activated through the RLR signaling pathway (34). While mitochondrial MAVS induces IFN-I production, peroxisomal MAVS is primarily responsible for IFN-III induction (35). Peroxisomes are now recognized as key subcellular platforms for antiviral signaling due to MAVS localization (12, 36). Similar to our findings, viruses, such as hepatitis C virus and human cytomegalovirus, exploit this system by targeting peroxisomal MAVS to block IFN-III responses (36–40), consistent with our findings.

Beyond MAVS localization, peroxisome abundance and plasticity are also critical for regulating immune signaling (41). For example, flaviviruses like dengue and West Nile virus reduce peroxisome numbers by targeting the peroxisomal biogenesis factor PEX19, leading to diminished IFN-III production (42). Odendall et al. also found that increasing peroxisome abundance enhances IFN-III expression (9). A prior study revealed that PEDV NSP1 suppresses IFN-III production by reducing peroxisome abundance (16). Consistent with this, our study revealed that both PEDV infection and NSP8 overexpression decreased the expression of peroxisome-associated proteins and overall peroxisome abundance. This loss is mediated via pexophagy, the selective autophagic degradation of peroxisomes. Supporting this, we demonstrated that PEDV infection and NSP8 overexpression both activate autophagy and pexophagy, which contribute to the suppression of MAVS-mediated IFN-III production.

To investigate the molecular mechanism by which NSP8 induces pexophagy, we performed mass spectrometry-based screening and identified PEX13 as a direct binding partner. This interaction was further confirmed through Co-IP and confocal microscopy assays. PEX13 is a key component of the peroxisomal matrix import machinery and plays a protective role against peroxisomal degradation. PEX13 can recognize proteins with the PTS1 signal and mediate their entry into peroxisomes (43). The absence of PEX13 leads to ER stress (44). Research has found that PEX13 serves as a novel factor regulating pexophagy, and reducing the protein level of PEX13 is an effective and intrinsic way to induce pexophagy (20). Loss of PEX13 leads to the accumulation of ubiquitinated PEX5 on peroxisomes, a hallmark trigger of pexophagy. Our findings revealed that both PEDV infection and NSP8 overexpression suppress PEX13 expression, and this downregulation occurs via the autophagy pathway. Other studies have found that during Porcine Deltacoronavirus (PDCOV) infection, the SIRT5 protein regulates the desuccinylation of the PDCOV M protein, which in turn leads to an increase in the ubiquitination of PEX5 and initiates pexophagy (45). Newcastle disease virus infection triggers excessive ROS production, activating the phosphorylation and peroxisomal localization of ataxia-telangiectasia mutated (ATM). Activated ATM promotes the interaction between the peroxisomal receptor PEX5, driving pexophagy (46). Consistent with this, further mechanistic studies revealed that NSP8 reduces the pool of PEX13-bound PEX5, facilitating its ubiquitination. Ubiquitinated PEX5 subsequently recruits the autophagy receptor NBR1, promoting autophagosome formation (20, 47). Notably, PEX2, an E3 ubiquitin ligase, is also involved in mediating PEX5 ubiquitination during pexophagy (30). We found that NSP8 enhances PEX5 ubiquitination and strengthens its interactions with both PEX2 and NBR1. Importantly, the NSP8-induced reduction in peroxisome abundance was abolished when PEX5, PEX2, NBR1, or ATG7 were silenced, highlighting their essential roles in this pathway. These findings establish that NSP8 induces pexophagy by downregulating PEX13, a novel regulator of peroxisomal autophagy. This disruption impairs MAVS-mediated signaling. Supporting this, NSP8-mediated suppression of MAVS was exacerbated in PEX13 knockdown cells but alleviated in PEX13-overexpressing cells. Furthermore, IFN-III expression was significantly reduced in PEX13-KD cells and elevated in PEX13-OE cells compared to wild-type controls.

**Fig 8:**
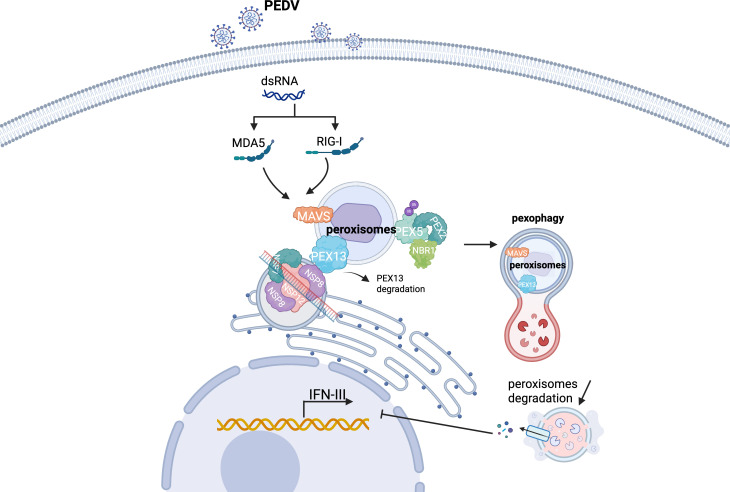
Schematic diagram illustrating the mechanism by which PEDV NSP8 induces pexophagy to suppress MAVS-mediated IFN-III production. PEDV NSP8 evades innate immunity by recruiting PEX13 and interacting with PEX13 to trigger its autophagy degradation, which promotes PEX5 ubiquitination and initiates pexophagy, ultimately leading to peroxisome degradation and suppression of MAVS-mediated IFN-III production.

In summary, our study reveals a previously unrecognized mechanism by which PEDV NSP8 hijacks the host autophagy machinery to degrade peroxisomes through a PEX13-dependent pexophagy pathway (Fig. 8). This process undermines peroxisomal MAVS signaling and suppresses IFN-III production, allowing PEDV to evade antiviral responses. These insights provide a potential basis for developing antiviral strategies targeting the NSP8–PEX13–pexophagy axis.

## MATERIALS AND METHODS

### Cell lines, viruses, and plasmids

Vero cells (ATCC CCL-81) and 293T cells (ATCC CRL-3216) were cultured in Dulbecco’s modified Eagle’s medium (DMEM) (Corning, USA) supplemented with 10% fetal calf serum (Gibco, USA) and 50 IU/mL penicillin–streptomycin (30-002-CI, Corning, USA). IPEC-J2, a continuous line of epithelial cells derived from the jejunum of a 12-h-old, colostrum-deprived, mixed breed piglet (48), was also maintained in DMEM (Corning, USA). LLC-PK1 cells (ATCC CL-101) were cultured in MEM (Corning, USA). All cells were incubated at 37°C in a humidified incubator with 5% CO_2_.

The PEDV variant strain MSCH (GenBank accession no. MT683617) was isolated and maintained in our laboratory. The virus was propagated in Vero cells and IPEC-J2 cells with 6 µg/mL trypsin (Sigma-Aldrich, USA) as previously described (49).

Plasmids used in this study included pIFN-λ1(−225 to −36), HA-PEX13, GFP-PEX13, MYC-NSP8, GFP-SKL, RFP-GFP-SKL, Flag-PEX5, and various NSP8 and PEX13 truncation constructs (all constructed in-house using standard molecular cloning procedures), except for GFP-NSP8-N3, which was synthesized by Tsingke Biotechnology (Beijing, China). Additional expression plasmids, including HA-RIG-I, HA-MDA5, HA-MAVS, HA-IRF3, Flag-TBK1, Flag-IKKε, HA-UB, and pRL-TK, were kindly provided by Dr. Xing Liu. Plasmids encoding PEDV proteins were generated as previously described (50).

### Antibodies and chemicals

Rabbit antibodies against LC3, P62, MAVS, catalase, PEX2, PMP70, and PEX13 were purchased from Proteintech (USA). Mouse antibodies against GFP and Flag were obtained from Abmart (China), while anti-NBR1 and anti-PEX5 antibodies were acquired from Santa Cruz Biotechnology (USA). Rabbit anti-HA and anti-ATG7 antibodies were sourced from Cell Signaling Technology (CST) (USA). Monoclonal antibodies against the PEDV N protein and polyclonal antibodies against NSP8 were preserved in our laboratory. Horseradish peroxidase (HRP)-conjugated secondary antibodies against rabbit or mouse IgG were obtained from Bioss (China). For chemical treatments, cells were incubated with DMSO, proteasome inhibitors, or autolysosome inhibitors, ensuring that the final DMSO concentration did not exceed 2%. The proteasome inhibitor MG132 and the autophagy inhibitors 3-methyladenine (3-MA) and bafilomycin A1 (Baf-A1) were purchased from Selleck (USA).

### Plasmids and molecular clones

Plasmid transfections in 293T cells were carried out using Lipofectamine 3000 (Thermo Fisher Scientific, L3000015), while transfections in Vero and IPEC-J2 cells were performed using Lipo8000 (Bryotime, C0533). For siRNA transfection, Lipofectamine 3000 was used to transfect siRNAs into IPEC-J2 cells following the manufacturer’s instructions. All transfection procedures were conducted in accordance with the recommended protocols provided by the respective manufacturers. The sequences of the sgRNAs and siRNAs used in this study are listed in Table 1.

**TABLE 1:** The sequences of sgRNA and siRNA used in this study

Primers	Sequences (5′–3′)	Sequences (5′–3′)
siPEX2	CUCUUACUGGUGCACCUAA	UUAGGUGCACCAGUAAGAGTT
siPEX5	CUGGCUUUCUGACUAUGAUTT	AUCAUAGUCAGAAAGCCAGTT
siNBR1-1	UCAGAGAGCAAGUGGUAAATT	UUUACCACUUGCUCUCUGATT
siNBR1-2	GGAAAUGUAAAGUGGAGUATT	UACUCCACUUUACAUUUCCTT
siNBR1-3	GGGAAGAGAUCGUCCAUAUTT	AUAUGGACGAUCUCUUCCCTT
siATG7-1	GCGAAUGUAUGGACCCUAATT	UUAGGGUCCAUACAUUCGCTT
siATG7-2	GCUAGGACGUUGAUGGGUUTT	AACCCAUCAACGUCCUAGCTT
siATG7-3	AGAGAAAGCUGGUCAUCAATT	UUGAUGACCAGCUUUCUCUTT
siNC	UUCUCCGAACGUGUCACGUTT	ACGUGACACGUUCGGAGAATT
sgPEX13	CACCGGGGAGACCCGCCGGATTCCG	AAACCGGAATCCGGCGGGTCTCCCC

### Peroxisome isolation

Peroxisome isolation was performed from IPEC-J2 cells using the Peroxisome Isolation Kit (Sigma, PEROX1). Briefly, 2 × 10⁸ cells were harvested, washed in phosphate-buffered saline, and centrifuged at 250 × g for 5 min. The cell pellet was resuspended in peroxisome extraction buffer [5 mM 3-(N-morpholino) propanesulfonic acid, pH 7.65, 0.25 M sucrose, 1 mM EDTA, 0.1% ethanol, and protease inhibitor cocktail], vortexed, and homogenized with a 7 mL Dounce homogenizer. The homogenate was centrifuged sequentially at 1,000 × *g* and 2,000 × *g* for 10 min each. The supernatant was then centrifuged at 25,000 × *g* for 20 min to isolate the crude peroxisomal fraction, which was further purified by density gradient centrifugation using Optiprep. After centrifugation at 100,000 × *g* for 1.5 h, the purified peroxisomes were collected for analysis.

### Western blotting analysis

Cells were lysed using radioimmunoprecipitation assay lysis buffer (Beyotime, China) on ice for 30 min. The lysates were separated by SDS-PAGE and subsequently transferred onto nitrocellulose membranes. Membranes were then blocked with 10% nonfat milk in PBST for 2 h at room temperature (RT), followed by washing with PBST and incubation with the appropriate primary antibodies overnight at 4°C. Afterward, membranes were incubated with HRP-conjugated secondary antibodies for 1 h at RT. Protein bands were visualized using an enhanced chemiluminescence kit (Tanon, China), and signal intensities were captured and analyzed using a Tanon 5200 chemiluminescence imaging system (Tanon, China).

### Quantitative real-time PCR

Cells were washed once with PBS, and total RNA was extracted using the Total RNA Kit I (Omega Bio-Tek, USA) according to the manufacturer’s instructions. For reverse transcription, 1 µg of RNA was reverse transcribed using HiScript qRT SuperMix (Vazyme, China). Quantitative PCR was conducted on an ABI QuantStudio 6 System (Applied Biosystems, USA) with AceQ qPCR SYBR Green Master Mix (Vazyme, China) following the provided instructions.

Relative gene expression was calculated using the comparative cycle threshold (CT) method, with GAPDH used as the internal control for normalization of both host and viral gene expression. The primers used in this study are listed in Table 2.

**TABLE 2:** The primers used in this study

Primer	Forward primer (5′–3′)	Reverse primer (5′–3′)
PEDV N	CGTACAGGTAAGTCAATTAC	GATGAAGCATTGACTGAA
β-Actin	CTCCATCATGAAGTGCGACGT	GTGATCTCCTTCTGCATCCTGTC
IFN-λ1	GGTGCTGGCGACTGTGATG	GATTGGAACTGGCCCATGTG
IFN-λ3	ACTTGGCCCAGTTCAAGTCT	CATCCTTGGCCCTCTTGA
IFN-λ4	GCTATGGGACTGTGGGTCTT	AGGGAGCGGTAGTGAGAGAG

### Lentivirus production and transduction

Lentiviral particles were produced by transient co-transfection of the packaging plasmids pMD2.G (Addgene #12259), psPAX2 (Addgene #12260), and plentiCRISPRv2-sgRNA into HEK293T cells using polyethylenimine transfection reagent (Yeasen, China). Medium was replaced 12 hours post-transfection (hpt), and viral supernatants were collected 48 and 60 hpt and stored at −80°C. Target cells were transduced with lentivirus in the presence of 10 µg/mL polybrene.

### Co-immunoprecipitation assay

For Co-IP, cells were transfected with plasmids for 24 h, lysed in NP40 cell buffer containing PMSF (protease inhibitor, Beyotime, China), and centrifuged. Supernatants were incubated with anti-Flag (Sigma, USA), anti-HA (CST, USA), or anti-GFP (Proteintech, USA) magnetic beads. Beads were washed with PBST and eluted in 50 mM glycine buffer (pH 2.8). The eluted proteins were analyzed by immunoblotting using appropriate antibodies.

### Confocal microscopy

For immunofluorescence, cells were cultured on 15 mm glass-bottom dishes (Nest Biotechnology, China), fixed with 4% paraformaldehyde for 10 min at RT, permeabilized with 0.1% Triton X-100 for 10 min, and blocked with 2% BSA in PBS for 1 h. Primary antibodies were applied overnight at 4°C, followed by incubation with Alexa Fluor 488-conjugated secondary antibodies (Proteintech, China) for 1 h at RT. Nuclei were then stained with DAPI (Biosharp, China) for 10 min. Fluorescence images were captured using a Nikon A1 confocal microscope (Japan), and two or three channels were recorded either sequentially or simultaneously while avoiding signal overlap.

### Dual luciferase assay

For the dual luciferase assay, 293T cells grown in 24-well plates were co-transfected with the IFN-λ1-Luc reporter plasmid, pRL-TK internal control plasmid, and various expression plasmids or control vectors using Lipofectamine 3000, according to the manufacturer’s instructions. At 36 hpt, cells were harvested and luciferase activity was measured using a dual-luciferase reporter assay kit according to standard procedures.

### Statistical analysis

Data from at least three independent experiments were analyzed using one-way or two-way analysis of variance followed by Tukey’s *post hoc* test for multiple comparisons (GraphPad Prism Software Inc., San Diego, CA, USA). Results are expressed as the mean ± SD (standard deviation). A *P* value of <0.05 was considered statistically significant (*), while *P* values of <0.01 (**), <0.001 (***), and <0.0001 (****) were considered highly significant.

## SUPPLEMENTAL MATERIAL

Figure S1 mbio.02396-25-s0001.tif The degradation of peroxisomes during PEDV infection via the autophagy pathway.

Figure S2 mbio.02396-25-s0002.tif PEDV NSP8 suppresses MAVS expression and inhibits IFN-Ⅲ production through the degradation of PEX13.

Legends mbio.02396-25-s0003.docx Legends for Fig. S1 and S2.

ASM does not own the copyrights to Supplemental Material that may be linked to, or accessed through, an article. The authors have granted ASM a non-exclusive, world-wide license to publish the Supplemental Material files. Please contact the corresponding author directly for reuse.
