# Supplementary figures and images for: PEDV NSP8 inhibits IFN-III production induced by MAVS through downregulation of PEX13
Source: mBio. 2025 Nov 4;16(12):e02396-25. doi: 10.1128/mbio.02396-25 (PMC12691671; doi:10.1128/mbio.02396-25)

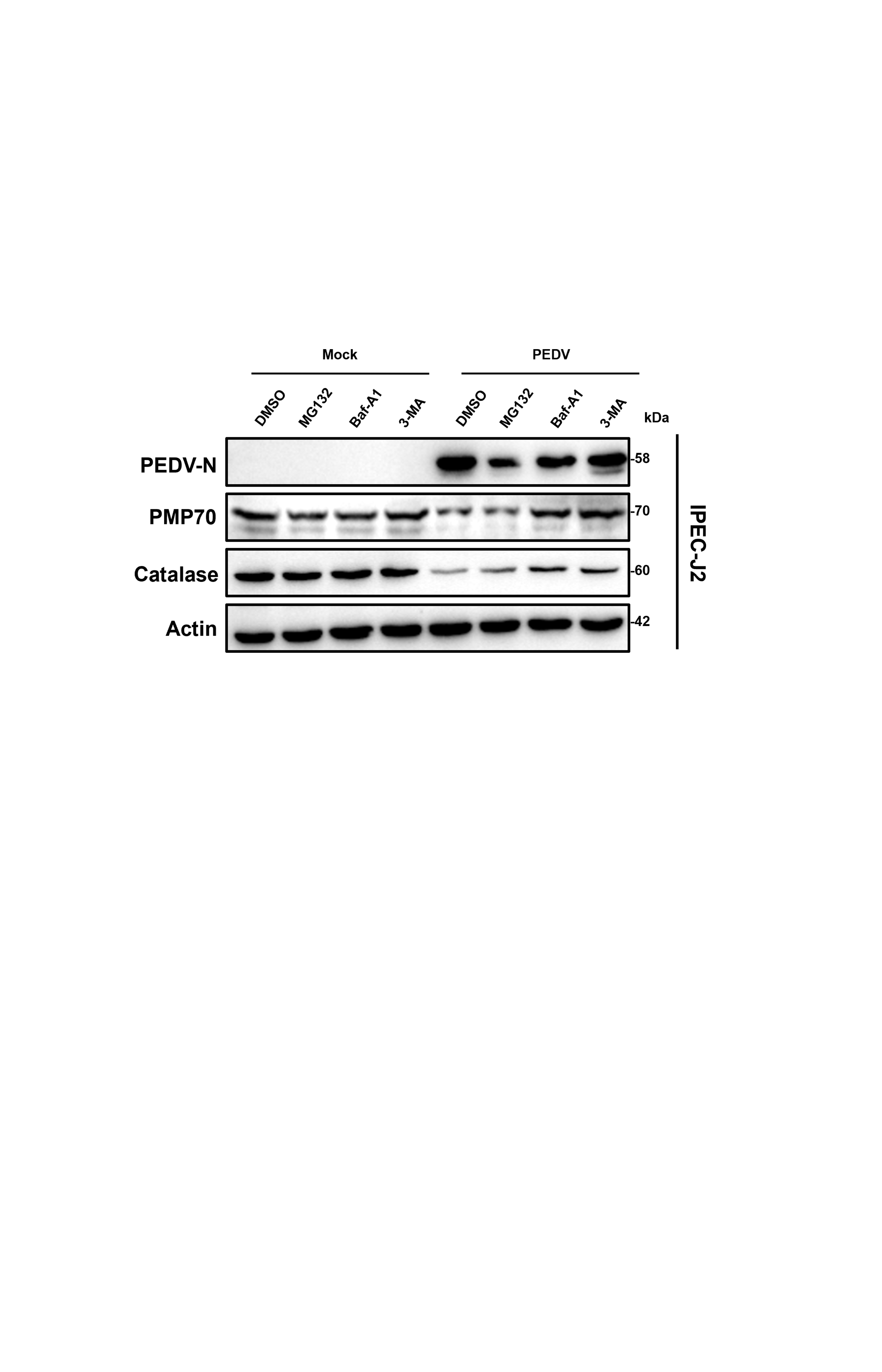

Supplement: Figure S1 — The degradation of peroxisomes during PEDV infection via the autophagy pathway. [file mbio.02396-25-s0001.tif]

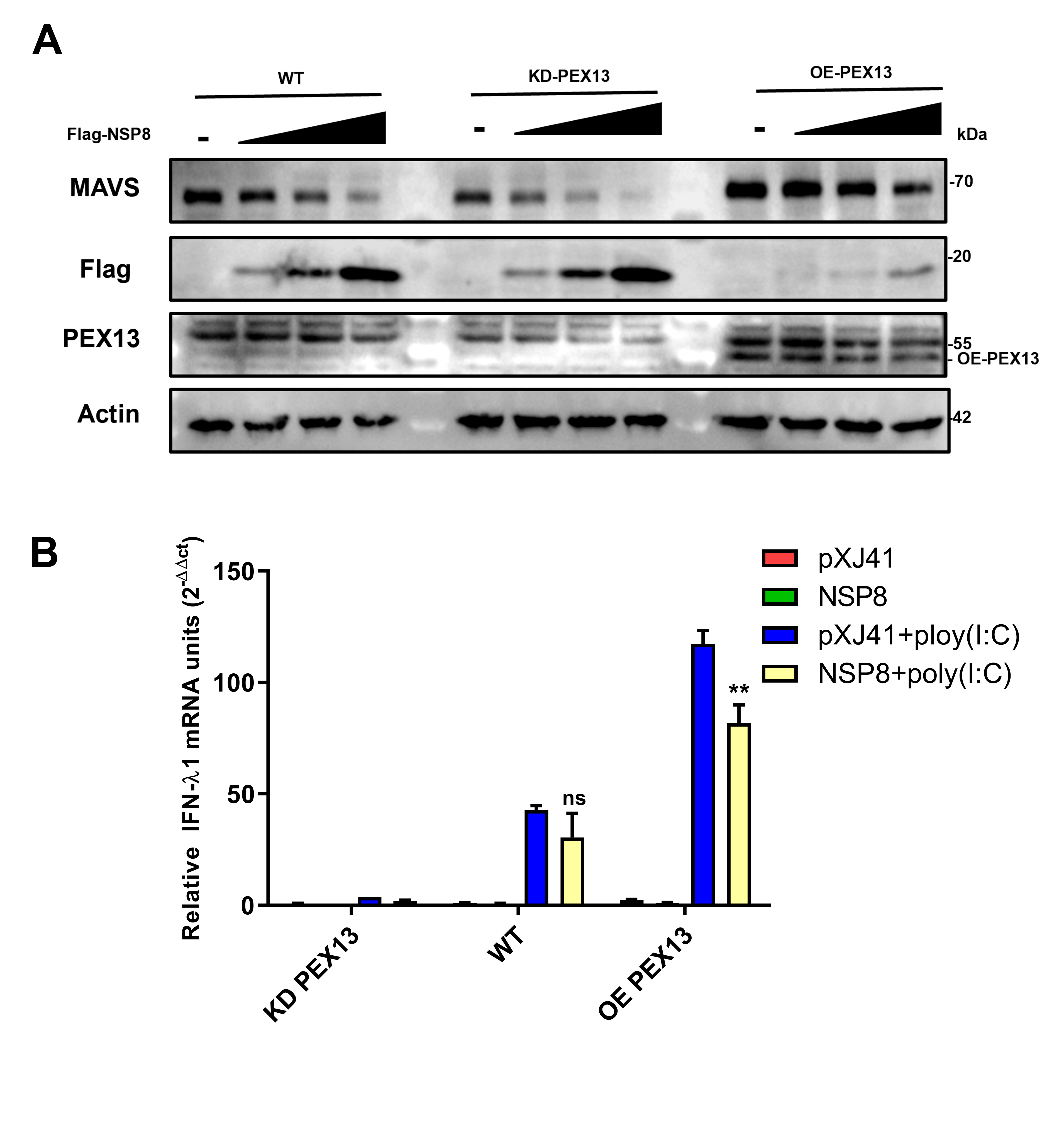

Supplement: Figure S2 — PEDV NSP8 suppresses MAVS expression and inhibits IFN-Ⅲ production through the degradation of PEX13. [file mbio.02396-25-s0002.tif]
