# Supplementary material for: PEDV NSP8 inhibits IFN-III production induced by MAVS through downregulation of PEX13
Source: mBio. 2025 Nov 4;16(12):e02396-25. doi: 10.1128/mbio.02396-25 (PMC12691671; doi:10.1128/mbio.02396-25)
Supplement: Legends — for Fig. S1 and S2. [file mbio.02396-25-s0003.docx]

**Fig. S1** The degradation of peroxisome during PEDV infection via autophagy pathway. IPEC-J2 cells seeded in 12-well plates were mock-infected or infected with PEDV. At 2 hpi, cells were treated with DMSO, MG132 (10 nM), Baf-A1 (25 nM), or 3-MA (2 mM). After an additional 22 h, cells were harvested for Western blotting analysis with antibodies against PEDV-N, PMP70, catalase, and actin.

**Fig. S2** PEDV NSP8 suppresses MAVS expression and inhibits IFN-Ⅲ production through the degradation of PEX13. (**A**) WT, KD PEX13, or OE PEX13 cells seeded in 12-well plates were transfected with pXJ41 or increasing doses of Flag-NSP8 (2, 2.5, or 3 μg). At 24 hpt, the cells were harvested for Western blotting analysis using antibodies against MAVS, PEX13, Flag-tag, and actin. (**B**) WT, KD PEX13, or OE PEX13 cells seeded in 24-well plates were transfected with pXJ41 or Flag-NSP8. At 24 hpt, cells were stimulated with poly(I:C) at a concentration of 0.5 μg/mL. After another 12 h, the cells were harvested for RNA extraction, and RT-qPCR was performed to detect the expression of IFN-λ1.
